# The Impact of Language Discordance on Acquiring Broad Social History: A Qualitative Study of Patients, Clinicians, and Interpreters

**DOI:** 10.1007/s11606-024-09234-3

**Published:** 2024-12-13

**Authors:** Bansri Doshi, Isabel Luna, Ruby Quesada, Maria E. Garcia, Alicia Fernandez, Elaine C. Khoong

**Affiliations:** 1 University of California, San Francisco (UCSF) School of Medicine, Berkeley, CA USA; 2 Joint Medical Program, University of California, Berkeley – UCSF, Berkeley, CA USA; 3 Division of General Internal Medicine, Department of Medicine, UCSF, San Francisco, CA USA; 4 UCSF Action Research Center for Health Equity, San Francisco, CA USA; 5 UCSF Health Interpreting Services, San Francisco, CA USA; 6 UCSF Department of Epidemiology & Biostatistics, San Francisco, CA USA; 7 UCSF Latinx Center of Excellence, San Francisco, CA USA

**Keywords:** language barriers, limited English proficiency, interpreter, social history, social determinants of health

## Abstract

**Background:**

By acquiring a broad social history (BSH), which includes aspects of patients’ social needs in addition to their hobbies and activities, values, and perspectives on care, clinicians can provide more patient-centered care, which is known to improve health outcomes. However, the impact of patient-clinician language discordance on acquisition of BSH has not been well studied.

**Objective:**

To ascertain whether language discordance impacts clinicians’ acquisition of patients’ BSH by interviewing patients, clinicians, and medical interpreters.

**Design:**

Qualitative study based on semi-structured interviews, followed by thematic analysis using rapid qualitative data analysis methods.

**Participants (or Patients or Subjects):**

We interviewed 8 Spanish-speaking and 5 English-speaking patients seen for primary care within a public hospital in San Francisco, 6 primary care clinicians, and 8 medical interpreters.

**Approach:**

All interviews were recorded and transcribed. The transcriptions were summarized into domains using rapid qualitative data analysis. The summaries were displayed in a matrix and used to generate themes and subthemes.

**Key Results:**

Analysis of interviews highlighted two overarching themes concerning BSH acquisition. One theme was the importance of BSH: namely, it is critical for patient-clinician relationships and personalized care. Commonly elicited BSH facets were social drivers of health, but leisure activities were less frequently asked. The second theme described ways in which language discordance, via the need for interpretation, affects the collection of BSH, both through increasing time pressure, and hindering rapport-building strategies. Cultural barriers pose an obstacle to BSH acquisition and may operate independently of language discordance.

**Conclusions:**

Our results suggest two action steps for health systems: (a) apportioning more time for language-discordant visits to increase rapport-building and BSH acquisition, and (b) increasing training for clinicians on acquiring BSH when working with interpreters. Further research is needed to investigate the relationship between language discordance, acquisition of BSH, and patient outcomes.

**Supplementary Information:**

The online version contains supplementary material available at 10.1007/s11606-024-09234-3.

## INTRODUCTION

Asking patients about their environment, family life, work, and relationships, often termed “taking a social history,” forms part of the bedrock of patient-centered communication and development of a patient-clinician relationship.^1^ Social history questions are generally described as gathering information on personal details that are clinically significant and may serve to improve the patient-clinician relationship, which has been shown to have a benefit on health outcomes.^2^

The traditional social history can include a wide range of topics, some of the most queried aspects being health-related behaviors (smoking, alcohol use, exercise, substance use) as well as topics such as family, social support, sexual history, and employment.^3^ In this study, we expanded the scope of social history to “broad social history (BSH),” which includes individualizing knowledge,^4^ or “knowledge of the patient as a person^5^”—encompassing patients’ values, beliefs, and perspectives on health.^6^

Acquisition of broad social history is critical to patient care for several reasons. BSH forms part of the foundation of patient-centered care,^7^ which is associated with decreased health care utilization.^8^ In patient-centered interactions, where the patients’ perspectives on their health—a facet of BSH—is elicited, patient satisfaction increases, as does adherence to recommendation,^9^ and better recovery.^10^ BSH collection that leads to the incorporation of patient-centered interventions has also been shown to improve health outcomes.^11,12^ When clinicians fail to acquire BSH, the quality of the patient-clinician relationship suffers, and clinicians may be more likely to misdiagnose, mistreat, and cause harm.^13^

BSH can be collected through various mechanisms. In addition to purposeful discussion during a clinical encounter, previous papers have described “topicalized small talk,^14^” as ways for clinicians to gather these pieces of information about their patients and build the patient-clinician relationship. Clinicians might also gather BSH information based on chart review,^15^ or the results of questionnaires administered outside of the patient-clinician encounter. Such questionnaires are often utilized in social needs screening (which largely screens for information about social drivers of health, or SDoH), and is being increasingly adopted by health centers. ^16,17^ However, while systematic screening surveys can inform clinicians’ knowledge about their patients’ SDoH, they may not replace the valuable information and rapport building that occurs when clinicians acquire BSH directly from their patients, either through purposeful questions or small talk.

Another factor that impacts patient-centered care delivery is the ability to communicate, which is impacted by language discordance (when patients and clinicians speak a different language). While it is known that language discordance negatively impacts health outcomes, the interplay between language discordance and acquisition of BSH is less known. Previous literature has explored the impact of language discordance on various aspects of patient care. Specifically, it leads to poorer health outcomes for non-English-language preferring (NELP) patients when trained medical interpreters are not present, and worse patient-clinician communication across multiple fields even when interpreters are present.^18–20^ Language discordance is also known to impact the delivery of patient-centered care, of which BSH is a critical component.^21^ According to both qualitative and quantitative studies, patients who experience language-discordant care experience fewer statements of partnership, empathetic responses, rapport establishment, less respectful communication, and less concern for the patient as a person from clinicians.^22–26^ The impact of language discordance on clinician acquisition of BSH is still being explored.^27^ Improving the ways in which clinicians acquire BSH in language-discordant encounters may lead to a more personalized knowledge of patients (i.e., patient individualization), and improve trust and communication in the patient-clinician relationship, which are both known to improve patient satisfaction and health outcomes.^28,29^

To address the gap in the literature on how language discordance impacts specifically the collection of BSH, we conducted semi-structured interviews with English and Spanish-speaking patients, clinicians, and medical interpreters. The central aims of this qualitative study were to explore (a) the perceived importance of BSH and participants’ experiences of obtaining BSH and (b) the process by which language discordance impacts clinicians’ ability to obtain BSH.

## METHODS

### Study Design

This was a qualitative study consisting of semi-structured individual interviews with Spanish- and English-speaking patients, primary care clinicians, and medical interpreters. All procedures were approved by the institutional review board (UCSF IRB 23–38822).

### Participant Recruitment and Eligibility

BD and IL recruited patients who received primary care at a safety-net hospital in San Francisco, CA, which treats a highly linguistically diverse population. Patients were recruited from a database of patients who had agreed to participate in previous interview-based studies at their respective clinics. They were eligible if they spoke English or Spanish and had received care from the same primary clinician at least three times (to get their perspective on how their PCP obtained a broad social history and built the patient-clinician relationship). Of note, we interviewed English-preferring patients to evaluate whether perspectives differed between patients who generally experience language concordance vs. discordance. BD, IL, RQ, and EK recruited clinicians and medical interpreters via email distribution to department heads. Eligible clinicians included physicians and NPs who practiced in primary care settings in San Francisco. Any medical interpreter employed by San Francisco Health Network or UCSF Health was eligible.

### Data Collection

BD, IL, RQ, MEG, and EK created four semi-structured interview guides for Spanish-speaking patients, English-speaking patients, clinicians, and interpreters. The interview guides were designed to understand the importance of contextualizing information and BSH to participants and to understand the processes by which language discordance impacts BSH acquisition. BD and IL pilot tested interview guides with three clinicians, four patients (two English-speaking; two Spanish-speaking), and one interpreter. Components of the interview guides (ESM Appendix) included: (a) how a patient-clinician relationship is built and what factors affect it; (b) social history components; and (c) how social history is obtained and what factors affect it. BD and IL conducted and recorded all interviews.

### Data Analysis

Interviews were transcribed in the language they were conducted (English or Spanish, according to the languages spoken by the interviewers BD and IL). The study team then analyzed de-identified interview transcripts using thematic analysis and rapid qualitative data analysis,^30^ in which transcripts were summarized into templates that contained domains for each key area. BD, IL, and RQ summarized Spanish interview transcripts in English, and RQ (a Spanish medical interpreter) translated key quotes. To establish guidelines for consistency and thoroughness in summary templates, the core study team (BD, IL, RQ, and EK) collectively reviewed two independently completed templates for one selected transcript of each of the four participant groups. The remaining transcripts underwent a double-coding process where one study team member summarized the content, and another independently reviewed each transcript and summary to triangulate viewpoints and ensure comprehensiveness. Interview summaries from each participant group (patients, clinicians, and medical interpreters) were imported into a matrix that displayed material in each domain (in columns) across all participants (in rows). BD and IL aggregated matrix components into memos, which were used to generate themes and subthemes. Themes were reviewed iteratively by the entire study team to resolve inconsistencies. The study team was comprised of a medical student (BD), a research data analyst (IL), a medical interpreter (RQ), and primary care physicians (MEG, AF, EK), which allowed for data, subthemes, and themes to be contextualized across multiple roles and viewpoints. In reporting the results, we followed the Standards for Reporting Qualitative Research (SRQR) guidelines.^31^

## RESULTS

This study included 13 patients (8 Spanish-language preferring patients, 5 English-language preferring patients), 6 primary care clinicians with varying degrees of Spanish proficiency, and 8 medical interpreters, at which point the study team concluded that thematic sufficiency had been reached as new themes and subthemes were not emerging (ESM Appendix Table 1).

Our clinicians, patients, and interpreters expressed similar perspectives. Therefore, we present our themes across all three groups. However, if themes or subthemes emerged for only some of the groups or if there were differing perspectives, we specify this below.

### Theme 1: Perspectives on a BSH and the Patient-Clinician Relationship (Representative Quotes in Table 1)

**Table 1:** Representative Quotes for Perspectives on BSH

Subtheme	Summary	Quote
*Importance of BSH*	BSH information can help build a better, more trusting relationship	“Muy importante compartir tu relación social, todo eso. ‘¿Cómo te va?’ Muy importante, para que el doctor me conozca más y yo tenerle más confianza.” “**It’s very** **important to share about your social relationships, and all that. ‘How’s it going?’ Very important, so that the doctor knows me better and I trust him more**.”—Patient 6 (Spanish), referring to a language-concordant interaction
		“I think it’s wonderful because…[my PCP] not only cares about me in a medical sense, but she’s going above and beyond to make sure I have housing, I have food, my kids have what they need. I find that awesome.” Patient 13 (English)
		“All those [BSH] issues are always addressed…living situation, transportation, everything… Patients are happy that even without them mentioning the issue, the question came up, and it was addressed too.”—Medical Interpreter 8 (Russian)
*Most asked aspects of BSH*	Clinicians ask a range of BSH questions	“A veces me pregunta acerca de mi país…sobre mis rutinas y las cosas que hacía. Entonces yo siento que eso es algo que no a todos los médicos les importa, no todos te preguntan algo así. Cuando nos vemos él siempre me pregunta ‘Hola como estas, que noticias hay de Chile.’” “**Sometimes he asks me about my country…about my routines and the things I did. So I feel these are not things all doctors care about, not all of them ask you things like that. When we see each other, he always asks me, ‘Hi, how are you, what news from Chile?**’”**—**Patient 7 (Spanish), referring to a language-concordant interaction
*How BSH is obtained*	Obtaining BSH questions directly (versus a questionnaire) is preferred	“No me gustaría que me lo preguntara por una encuesta, me gustaría que me lo preguntara cuando llegue a consulta…Me gustaría sentir que aunque el doctor tenga que hacer esto como protocolo, me gustaría sentir que no es un protocol…no por protocolo sino porque realmente se interesa por mí.” “**I wouldn’t like to be asked on a survey, I would like to be asked when I arrive for the appointment…I would like to feel that, even though the doctor has to ask for protocol, I’d like to feel that it wasn’t protocol…not for protocol but because he actually is interested about me**.”—Patient 7 (Spanish), referring to a language-concordant interaction

#### Importance of BSH

All three participant groups considered acquisition of a BSH crucial for establishing rapport and building trust. Clinicians expressed its importance for personalizing patient care. Medical interpreters reflected on experiences with patients and shared their perception that patients generally appreciate it when clinicians ask questions around BSH. Patients especially reported feeling cared for and better understood when clinicians asked about their families, social lives, and cultures. Some patients expressed the importance of clinicians asking them about their mental health and addressing stigmatization related to having multiple marginalized identities.

#### Most and Least Asked Aspects of BSH

Clinicians and medical interpreters highlighted that a patient’s chief concern or problem list may impact what BSH questions are asked at a given visit. All participant groups reported that the factors they recalled most asking or being asked about were patients’ living situations, trauma/abuse, and social support. Among the least asked were hobbies and activities, unless clinicians viewed those topics as relevant to why the patient sought care or how they perceived their care.

#### How BSH Is Obtained

All participants reported that clinicians generally obtain BSH by asking patients direct questions, which was preferred by patients over paper or virtual questionnaires. Participants across groups expressed that patients sometimes initiate conversations about BSH if they were not asked these questions directly. When it came to who asks BSH questions, all participant groups mentioned that other staff are often instrumental in obtaining a BSH, especially medical assistants, nurses, and social workers.

### Theme 2: Language Discordance and BSH (Representative Quotes in Table 2)

**Table 2:** Representative Quotes for Impact of Language Discordance on SH

Subtheme	Summary	Quote
*Time pressure*	Time pressure in interpreted encounters cause deprioritization of BSH	“When you have an interpreter, the interaction is going to take at least twice as long. So that that can pose a barrier. It could mean that things that would I normally be able to do in a language concordant situation, I, you know, may need to prioritize what I do and leave some things undone for the next time.”—Clinician 1
		“Cuando ya tengo claro que esa cita se va a reducir a 15 minutos porque no hablo el idioma, tengo que enfocarme en mi prioridad que son los síntomas.” “**When it’s clear to me that the appointment is going to be reduced to 15 min because I don’t speak the language, I have to focus on my priority which are the symptoms**.”—Patient 8 (Spanish), referring to a language-discordant interaction
*Rapport-building & language discordance*	Rapport-building can feel awkward when interpreted, impeding natural approaches to obtaining BSH	“If I just ask a casual shoot-the-breeze question, it’s not really worth it to go through that with an interpreter. So, it definitely keeps it a level more formal.”—Clinician 4
*Cultural barriers*	Cultural barriers may form a barrier to obtaining BSH	“If an interpreter is doing their job correctly and…professionally, there should not be any language barriers. Period. What you probably mean [by language barriers] are cultural barriers, which language itself, may not cover.”—Medical Interpreter 3 (Spanish)
*Using interpretation to acquire BSH*	Interpretation may feel difficult for exploring nuanced BSH topics	“I think some of these questions are a little bit easier to get out there if is a straightforward answer, to sort of get it through an interpreter, yes/no, which is great for checkbox reasons, but maybe not so helpful for the patient…there’s just more barriers to exploring that. ‘Okay, they said they’re retired, but are they happy, or they’re not happy about that? What are they doing during their retirement, or are they safe? Are they financially solvent?’ It’s harder to just tell through an interpreter. Those are harder to explore unless you’re really intentional about it.”—Clinician 5

#### Increased Feeling of Time Pressure

Participants reported more time pressure in language-discordant encounters due to the use of interpretation services, resulting in deprioritizing BSH questions and focusing on questions perceived as most salient to the medical concern. Primary care clinicians reported booking additional appointments with language-discordant patients to discuss BSH. Clinicians also reported asking fewer open-ended questions in favor of saving time to address the top concerns on the patients’ problem lists. Patients shared similar concerns, reporting that regardless of setting, there was never enough time for clinicians to ask BSH questions, or thoroughly address their concerns and perspectives. Patients reported feeling conscious of language discordance increasing time pressure within the visit, thus leading them to focus on whatever their symptoms of highest priority were.

#### Rapport-Building and Language Discordance

Participants reported challenges in rapport-building in language-discordant interactions, which impacted their ability to gain BSH. Rapport-building strategies such as small talk, which can be informal strategies to segue into asking BSH questions, were less frequently employed. Clinicians described interpreted visits as adding a level of formality to the conversation. Some patients viewed it as a shared duty between clinicians and patients to surmount language discordance when it came to rapport-building. Some patients, being conscious of the impact language discordance could have on rapport-building, described making efforts to build rapport by pre-planning jokes and small talk. Clinicians and some medical interpreters expressed that rapport-building strategies, such as small talk, expressing empathy, or telling jokes, can feel awkward for clinicians in language-discordant settings due to a fear of appearing inauthentic, inexperience working with interpreters, and concerns about content not translating properly.

#### Cultural Barriers

Some medical interpreters expressed that when patients or clinicians are working with a certified medical interpreter, language barriers should be eliminated or limited, with the primary barrier being cultural discordance. Interpreters also emphasized their role as cultural brokers to bridge barriers in the encounter and address patient and provider questions. They also stated that clinicians can address cultural discordance by asking more questions pertaining to cultural factors, a perspective also shared by patients. Medical interpreters emphasized the need to increase clinician training around working with interpreters to acquire BSH in a culturally humble manner, and specifically to encourage clinicians to ask about cultural differences.

#### Interpretation and BSH

Trust in interpretation was described as a crucial component of clinicians’ ability to obtain BSH. Patients, interpreters, and clinicians highlighted factors that contributed to trust in interpretation, including familiarity with English or the interpreted language. Patients also reported a fear of being misunderstood and related instances of misunderstanding in the past due to language discordance and errors in interpretation. Interpreters particularly mentioned that it becomes easier to acquire BSH when patients and their loved ones trust that their interpretation is correct and non-judgmental.

Clinicians, interpreters, and some patients added that interpretation modalities in which facial expression or body language was easily seen increased accuracy and enhanced the ability to ask BSH questions through a greater feeling of inter-connectedness. Some participants in the clinician and medical interpreter groups held a different view regarding modalities and their impact on BSH, explaining that the skill and training of medical interpreters supersede the importance of interpretation modality; specifically, all things being equal an in-person interpreter was preferred. However, if the more accurate, trained interpreter was through telephone (e.g., a certified medical interpreter) that would be preferred over an inaccurate, untrained in-person interpreter (e.g., family members).

## DISCUSSION

### Findings

In this qualitative study of 27 interviews, patients, clinicians, and interpreters described BSH as a crucial aspect of patient care and the clinician-patient relationship, and that rapport-building and trust help clinicians obtain BSH. Patients preferred direct questioning by clinicians to acquire BSH, as it felt less perfunctory and enhanced the patient-clinician relationship. Across participant groups, patients were less likely to be asked about hobbies and activities (i.e., leisure). Language discordance was found to increase the feeling of time pressure in encounters due to interpretation, leading to the deprioritization of BSH. Interpretation was also identified as a potential barrier to building rapport and acquiring BSH, though trust in high-quality interpretation enhances the ability to obtain BSH. Interpreters highlighted that cultural barriers may also impede acquisition of BSH; however, participants suggested that clinicians can mitigate this by consciously asking more questions to gain insight into a patient’s cultural background and personal perspectives.

Our findings on the perceived importance of BSH acquisition on patient care and barriers imposed by language discordance are consistent with prior research. Providers’ acquisition of BSH factors such as social drivers of health and personalizing details are known to be extremely important to patients,^32^ and improve patient care and health outcomes.^33–35^ The finding that patients are infrequently asked about leisure activities reflects prior study findings that patients do not feel that physicians ask them questions to get to know them better.^36,37^ Our findings also underscore previous literature which has documented the negative impact that language discordance can have on patient-centered communication, time pressure, rapport-building, and screening patients for facets of BSH.^38–40^ In particular, time pressure has been understood to be one of the most reported reasons for choosing not to utilize medical interpreters.^41,42^

Our findings add to existing literature by collecting insights from all participants engaged in a language discordant encounter and exploring additional links between language discordance and BSH. Participants across groups directly linked feeling increased time pressures in language discordant encounters to the deprioritization of BSH questions. They also named feeling an added level of formality during interpreted encounters resulting in barriers to making small talk or jokes. Clinicians also added that the fear of sounding awkward or inauthentic hinders rapport-building strategies in interpreted encounters. Participants identified having trust in interpretation, some familiarity with the other language, or being familiar with navigating BSH questions in the context of interpreted encounters, as factors that facilitated the acquisition of BSH. Participants, particularly interpreters, emphasized the importance of mitigating cultural barriers and considering them separate from language discordance. A suggested mitigation strategy for clinicians was to display curiosity about patients’ cultures and being conscious of cultural factors when providing care recommendations.

### Clinical Implications

Our findings suggest key action steps for health systems, which will allow clinicians to overcome the barriers posed by language discordance on acquiring BSH. One step is apportioning more time in language-discordant encounters in order to reduce the perceived burden of time pressure from interpretation and to allow clinicians and patients greater time to discuss relevant individual beliefs, values, and experiences. Lengthening the time for clinicians to ask BSH questions in language-discordant encounters (or allowing more time for clinicians to review social needs screeners with language-discordant patients) will allow clinicians to create plans that incorporate more facets of the patient’s personal context and priorities. Another action step for health systems is to provide clinicians additional training on working with interpreters, with particular foci on diminishing clinicians’ feelings of awkwardness, inauthenticity, or increased formality. This training will encourage clinicians to engage in the same rapport-building strategies and acquisition of a broad social history as they would with a language-concordant patient.

### Strengths and Limitations

One key strength of this study is that it incorporates the perspectives of English- and Spanish-speaking patients, primary care clinicians, and medical interpreters. The inclusion of these perspectives in the aspects of language discordance and patient-clinician communication strengthens the findings and adds additional nuance to the literature.

One limitation of this study was a lack of language diversity among the patient participants, due to the languages spoken by the study team members in charge of interviews (English and Spanish). Therefore, there were no patient opinions to contextualize the perspectives of medical interpreters who spoke Mandarin, Cantonese, or Russian. Another limitation of this study is that racial and ethnic patient-clinician concordance was not queried, which on its own can affect patient-clinician communication.^43^

## CONCLUSIONS

In this qualitative study of 27 interviews spanning English- and Spanish-speaking patients, medical interpreters, and primary care clinicians, we found that language discordance impacts how clinicians obtain BSH about their patients by increasing the sense of time pressure due to the need for interpretation and adding a level of formality through interpretation that impacts clinicians’ ability to build rapport. We found that trust in interpretation, and familiarity in working with interpreters, can enhance clinicians’ ability to acquire BSH. We also found that cultural barriers affect BSH acquisition independently of language discordance and necessitates clinician training to mitigate cultural barriers.

Our results indicate that health systems should apportion more time for language-discordant visits to allow for more rapport-building and BSH questions. Health systems should also train clinicians to work effectively with interpreters (particularly to diminish the feeling of awkwardness or inauthenticity they may feel when using rapport-building strategies, and to encourage efforts to learn about patients’ cultures, values, and family backgrounds) to mitigate the effects of language and cultural discordance on BSH acquisition. There is also an opportunity for future research to build on these findings through an empiric analysis of recorded language-concordant or language-discordant PCP encounters to assess the frequency and type of BSH questions.

## Supplementary Information

Below is the link to the electronic supplementary material.

Supplementary file1 (DOCX 39 KB)

## References

[1] **Anderson R (Andy), Schiedermayer D.** The Social History Matters! *Acad Med*. 2010;85(7):1103. 10.1097/ACM.0b013e3181e19330 20592499 10.1097/ACM.0b013e3181e19330

[2] **Kelley JM, Kraft-Todd G, Schapira L, Kossowsky J, Riess H.** The influence of the patient-clinician relationship on healthcare outcomes: a systematic review and meta-analysis of randomized controlled trials. *PloS One*. 2014;9(4):e94207. 10.1371/journal.pone.0094207 24718585 10.1371/journal.pone.0094207 PMC3981763

[3] **Walsh C, Elhadad N.** Modeling Clinical Context: Rediscovering the Social History and Evaluating Language from the Clinic to the Wards. *AMIA Summits Transl Sci Proc*. 2014;2014:224-231. 25717417 PMC4333691

[4] **Grover S, Fitzpatrick A, Azim FT, et al.** Defining and implementing patient-centered care: An umbrella review. *Patient Educ Couns*. 2022;105(7):1679-1688. 10.1016/j.pec.2021.11.004 34848112 10.1016/j.pec.2021.11.004

[5] **Howe LC, Leibowitz KA, Crum AJ.** When Your Doctor “Gets It” and “Gets You”: The Critical Role of Competence and Warmth in the Patient–Provider Interaction. *Front Psychiatry*. 2019;10:475. 10.3389/fpsyt.2019.00475 31333518 10.3389/fpsyt.2019.00475 PMC6619399

[6] **Behforouz HL, Drain PK, Rhatigan JJ.** Rethinking the Social History. *N Engl J Med*. 2014;371(14):1277-1279. 10.1056/NEJMp1404846 25271600 10.1056/NEJMp1404846

[7] **Chapman BP, Duberstein PR, Epstein R, Fiscella K, Kravitz RL.** Patient Centered Communication During Primary Care Visits for Depressive Symptoms. *Med Care*. 2008;46(8):806-812. 10.1097/MLR.0b013e31817924e4 18665060 10.1097/MLR.0b013e31817924e4 PMC2727735

[8] **Bertakis KD, Azari R.** Patient-Centered Care is Associated with Decreased Health Care Utilization. *J Am Board Fam Med*. 2011;24(3):229-239. 10.3122/jabfm.2011.03.100170 21551394 10.3122/jabfm.2011.03.100170

[9] **Stewart MA.** What is a successful doctor-patient interview? A study of interactions and outcomes. *Soc Sci Med 1982*. 1984;19(2):167-175. 10.1016/0277-9536(84)90284-3 10.1016/0277-9536(84)90284-3 6474233

[10] **Stewart M, Brown JB, Donner A, et al.** The impact of patient-centered care on outcomes. *J Fam Pract*. 2000;49(9):796-804. 11032203

[11] **Rees S, Williams A.** Promoting and supporting self-management for adults living in the community with physical chronic illness: A systematic review of the effectiveness and meaningfulness of the patient-practitioner encounter. *JBI Libr Syst Rev*. 2009;7(13):492-582. 10.11124/01938924-200907130-00001 27819974 10.11124/01938924-200907130-00001

[12] **Kuntz JL, Safford MM, Singh JA, et al.** Patient-centered interventions to improve medication management and adherence: a qualitative review of research findings. *Patient Educ Couns*. 2014;97(3):310-326. 10.1016/j.pec.2014.08.021 25264309 10.1016/j.pec.2014.08.021 PMC5830099

[13] **Holmes SM, Hansen H, Jenks A, et al.** Misdiagnosis, Mistreatment, and Harm — When Medical Care Ignores Social Forces. *N Engl J Med*. 2020;382(12):1083-1086. 10.1056/NEJMp1916269 32187466 10.1056/NEJMp1916269

[14] **Hudak PL, Maynard DW.** An interactional approach to conceptualising small talk in medical interactions. *Sociol Health Illn*. 2011;33(4):634-653. 10.1111/j.1467-9566.2011.01343.x 21545445 10.1111/j.1467-9566.2011.01343.x PMC3609551

[15] **Gottlieb L, Tobey R, Cantor J, Hessler D, Adler NE. **Integrating Social And Medical Data To Improve Population Health: Opportunities And Barriers. *Health Aff (Millwood)*. 2016;35(11):2116-2123. 10.1377/hlthaff.2016.0723 27834254 10.1377/hlthaff.2016.0723

[16] **Tong ST, Liaw WR, Kashiri PL, et al.** Clinician Experiences with Screening for Social Needs in Primary Care. *J Am Board Fam Med JABFM*. 2018;31(3):351-363. 10.3122/jabfm.2018.03.170419 29743219 10.3122/jabfm.2018.03.170419 PMC6497466

[17] **Byhoff E, De Marchis EH, Hessler D, et al.** Part II: A Qualitative Study of Social Risk Screening Acceptability in Patients and Caregivers. *Am J Prev Med*. 2019;57(6 Suppl 1):S38-S46. 10.1016/j.amepre.2019.07.016 31753278 10.1016/j.amepre.2019.07.016 PMC6876708

[18] **Karliner LS, Jacobs EA, Chen AH, Mutha S.** Do Professional Interpreters Improve Clinical Care for Patients with Limited English Proficiency? A Systematic Review of the Literature. *Health Serv Res*. 2007;42(2):727-754. 10.1111/j.1475-6773.2006.00629.x 17362215 10.1111/j.1475-6773.2006.00629.x PMC1955368

[19] **Cano-Ibáñez N, Zolfaghari Y, Amezcua-Prieto C, Khan KS.** Physician–Patient Language Discordance and Poor Health Outcomes: A Systematic Scoping Review. *Front Public Health*. 2021;9:629041. 10.3389/fpubh.2021.629041 33816420 10.3389/fpubh.2021.629041 PMC8017287

[20] **Chu JN, Wong J, Bardach NS, et al.** Association between language discordance and unplanned hospital readmissions or emergency department revisits: a systematic review and meta-analysis. *BMJ Qual Saf*. Published online December 30, 2023. 10.1136/bmjqs-2023-016295 38160059 10.1136/bmjqs-2023-016295 PMC11186734

[21] **Karliner LS, Hwang ES, Nickleach D, Kaplan CP.** Language barriers and patient-centered breast cancer care. *Patient Educ Couns*. 2011;84(2):223-228. 10.1016/j.pec.2010.07.009 20685068 10.1016/j.pec.2010.07.009

[22] **Bregio C, Finik J, Baird M, et al.** Exploring the Impact of Language Concordance on Cancer Communication. *JCO Oncol Pract*. 2022;18(11):e1885-e1898. 10.1200/OP.22.00040 36112970 10.1200/OP.22.00040 PMC9653203

[23] **Rivadeneyra R, Elderkin-Thompson V, Silver RC, Waitzkin H.** Patient centeredness in medical encounters requiring an interpreter. *Am J Med*. 2000;108(6):470-474. 10.1016/s0002-9343(99)00445-3 10781779 10.1016/s0002-9343(99)00445-3

[24] **Baker DW, Hayes R, Fortier JP.** Interpreter Use and Satisfaction With Interpersonal Aspects of Care for Spanish-Speaking Patients. *Med Care*. 1998;36(10):1461. 9794340 10.1097/00005650-199810000-00004

[25] **Ferguson WJ, Candib LM.** Culture, language, and the doctor-patient relationship. *Fam Med*. 2002;34(5):353-361. 12038717

[26] **Schenker Y, Karter AJ, Schillinger D, et al.** The impact of limited English proficiency and physician language concordance on reports of clinical interactions among patients with diabetes: the DISTANCE Study. *Patient Educ Couns*. 2010;81(2):222-228. 10.1016/j.pec.2010.02.005 20223615 10.1016/j.pec.2010.02.005 PMC2907435

[27] **Litzau M, Turner J, Pettit K, Morgan Z, Cooper D.** Obtaining History with a Language Barrier in the Emergency Department: Perhaps not a Barrier After All. *West J Emerg Med*. 2018;19(6):934-937. 10.5811/westjem.2018.8.39146 30429924 10.5811/westjem.2018.8.39146 PMC6225939

[28] **Jasemi M, Valizadeh L, Zamanzadeh V, Keogh B.** A Concept Analysis of Holistic Care by Hybrid Model. *Indian J Palliat Care*. 2017;23(1):71-80. 10.4103/0973-1075.197960 28216867 10.4103/0973-1075.197960 PMC5294442

[29] **Yu C, Xian Y, Jing T, et al.** More patient-centered care, better healthcare: the association between patient-centered care and healthcare outcomes in inpatients. *Front Public Health*. 2023;11. 10.3389/fpubh.2023.1148277 37927879 10.3389/fpubh.2023.1148277 PMC10620693

[30] **Taylor B, Henshall C, Kenyon S, Litchfield I, Greenfield S.** Can rapid approaches to qualitative analysis deliver timely, valid findings to clinical leaders? A mixed methods study comparing rapid and thematic analysis. *BMJ Open*. 2018;8(10):e019993. 10.1136/bmjopen-2017-019993 30297341 10.1136/bmjopen-2017-019993 PMC6194404

[31] **O’Brien BC, Harris IB, Beckman TJ, Reed DA, Cook DA.** Standards for reporting qualitative research: a synthesis of recommendations. *Acad Med J Assoc Am Med Coll*. 2014;89(9):1245-1251. 10.1097/ACM.0000000000000388 10.1097/ACM.0000000000000388 24979285

[32] **Zimmerman DL, Min DJ, Davis-Collins A, DeBlieux P.** Treating Patients As People: What Do Hospital Patients Want Clinicians to Know About Them As a Person? *J Patient Exp*. 2020;7(2):270-274. 10.1177/2374373519826244 32851151 10.1177/2374373519826244 PMC7427369

[33] **Naz A, Rosenberg E, Andersson N, Labonté R, Andermann A**, CLEAR Collaboration. Health workers who ask about social determinants of health are more likely to report helping patients: Mixed-methods study. *Can Fam Physician Med Fam Can*. 2016;62(11):e684-e693. PMC9844577 28661888

[34] **Gottlieb LM, Wing H, Adler NE.** A Systematic Review of Interventions on Patients’ Social and Economic Needs. *Am J Prev Med*. 2017;53(5):719-729. 10.1016/j.amepre.2017.05.011 28688725 10.1016/j.amepre.2017.05.011

[35] **Yan AF, Chen Z, Wang Y, et al.** Effectiveness of Social Needs Screening and Interventions in Clinical Settings on Utilization, Cost, and Clinical Outcomes: A Systematic Review. *Health Equity*. 2022;6(1):454-475. 10.1089/heq.2022.0010 35801145 10.1089/heq.2022.0010 PMC9257553

[36] **Ziegelstein RC.** Perspectives in Primary Care: Knowing the Patient as a Person in the Precision Medicine Era. *Ann Fam Med*. 2018;16(1):4-5. 10.1370/afm.2169 29311168 10.1370/afm.2169 PMC5758313

[37] Unmet expectations: Doctors, patients give each other mixed reviews - amednews.com. January 29, 2007. Accessed October 11, 2024. https://amednews.com/article/20070129/profession/301299967/2/

[38] **Hsieh E.** Not Just “Getting by”: Factors Influencing Providers’ Choice of Interpreters. *J Gen Intern Med*. 2015;30(1):75-82. 10.1007/s11606-014-3066-8 25338731 10.1007/s11606-014-3066-8 PMC4284281

[39] **Ng J, Popova S, Yau M, Sulman J.** Do culturally sensitive services for Chinese in-patients make a difference? *Soc Work Health Care*. 2007;44(3):129-143. 10.1300/J010v44n03_01 17548271 10.1300/J010v44n03_01

[40] **Savitz ST, Nyman MA, Kaduk A, Loftus C, Phelan S, Barry BA.** Association of Patient and System-Level Factors With Social Determinants of Health Screening. *Med Care*. 2022;60(9):700-708. 10.1097/MLR.0000000000001754 35866557 10.1097/MLR.0000000000001754

[41] **Diamond LC, Schenker Y, Curry L, Bradley EH, Fernandez A.** Getting By: Underuse of Interpreters by Resident Physicians. *J Gen Intern Med*. 2009;24(2):256-262. 10.1007/s11606-008-0875-7 19089503 10.1007/s11606-008-0875-7 PMC2628994

[42] **Khoong EC, Fernandez A.** Addressing Gaps in Interpreter Use: Time for Implementation Science Informed Multi-Level Interventions. *J Gen Intern Med*. 2021;36(11):3532-3536. 10.1007/s11606-021-06823-4 33948799 10.1007/s11606-021-06823-4 PMC8606497

[43] **Shen MJ, Peterson EB, Costas-Muñiz R, et al.** The Effects of Race and Racial Concordance on Patient-Physician Communication: A Systematic Review of the Literature. *J Racial Ethn Health Disparities*. 2018;5(1):117-140. 10.1007/s40615-017-0350-4 28275996 10.1007/s40615-017-0350-4 PMC5591056

