# Supplementary material for: The Impact of Language Discordance on Acquiring Broad Social History: A Qualitative Study of Patients, Clinicians, and Interpreters
Source: J Gen Intern Med. 2024 Dec 13;40(8):1836–43. doi: 10.1007/s11606-024-09234-3 (PMC12119441; doi:10.1007/s11606-024-09234-3)
Supplement: Supplementary file 1 — Supplementary file1 (DOCX 39 KB) [file 11606_2024_9234_MOESM1_ESM.docx]

**Appendix**

***Please note:***

***Grayed-out questions are those that have been removed from thematic analysis of this study.***

***Contextual understanding is used to refer to a “Broad Social History.”***

**Appendix A: Interview Guides**

*Appendix A1: Spanish-speaking patient interview guide*

1. Introducción de entrevistador/a y participante *(Introduction of interviewer and participant)*
2. Descripción general del estudio y los objetivos centrales *(Brief overview of the study and the central aims)*

Hacemos este estudio para aprender que es lo que los pacientes esperan de sus doctores para desarrollar una relación doctor-paciente buena y confiable. Queremos enfocarnos en los factores que influyen la relación medico-paciente, y el impacto de compartir o no compartir el idioma con su doctor. *(We are doing this study to learn more about what patients expect from doctors to develop a trusting relationship. We want to focus on the factors that may influence the doctor-patient relationship, and the impact of sharing or not sharing a language with your doctor.)*

Preguntas: *(Questions)*

1. ¿Cuales son las características de una buena relación entre doctor y paciente? *(What are the characteristics of a good doctor-patient relationship?)*

Probe: ¿Cómo es la relación con su medico/a de cabecera? ¿Es una relación buena o mala, y por que? *(How is your relationship with your primary care doctor? Is it a good or bad relationship, and why?)*

Probe: Si hay algo que pudiera mejorar, ¿que sería? *(If there was something that could be improved, what would it be?)*

2. Ud. piensa que compartir el mismo idioma con su doctor cambia la relación? Cómo cambia? *(Do you think that sharing a language with your doctor changes the relationship? How?)*

Probe: If they have had language concordant or discordant doctors, and didn’t answer question 2 directly: Por favor, compare las relaciones que Usted ha tenido con doctores hispanohablantes y doctores no hispanohablantes. ¿Fueron diferentes? Cómo? *(Please compare the relationships you have had with Spanish-speaking and non-Spanish-speaking doctors. Were they different? How?)*

3. If patient mentions having had a visit w/interpreter: La ultima vez que le atendió un doctor que no hablaba espanol, ¿estaba usando un interprete? En caso de si, ¿que modo? Usaron un interprete por telefono, por videollamada, o uno que estaba ahi en persona? *(The last time you were seen by a non-Spanish-speaking doctor, were they using an interpreter? If yes, what type/mode? Did they use an phone, video, or in-person interpreter?)*

Probe: ¿Ud. piensa que el modo de interpretación (o traducción) cambia la relación con su doctor? *(Do you think that the mode of interpretation changes the relationship with your doctor?)*

Probe: ¿Que modo prefiere Ud.? *(What mode/type do you prefer?)*

Probe: ¿Le gustaría Ud. agregar algo mas sobre la interpretación/traducción? *(Would you like to add anything else about interpretation?)*

4. ¿Se siente ahorita que su doctor le conoce bien? *(Do you feel that your doctor knows you well?)*

¿Que señala que su doctor esta haciendo esfuerzas para conocerle bien? *(What signals that your doctor makes an effort to get to know you well?)*

5. ¿Su doctor de cabecera toma el tiempo para charlar o platicar con usted sobre la vida cotidiana? (En caso de si) ¿De que platican? *(Does your PCP take the time to chat with you about day-to-day life? [If yes] what about?)*

Probe: ¿Ud. piense que hacer tiempo para platicar sobre cosas de la vida diaria mejora, o no mejora, la relacion con su doctor de cabecera? *(Do you think that making time to chat about daily life improves, or does not improve, the relationship with your PCP?)*

Probe: En el caso de que haya tenido un doctor con el que no comparta el mismo idioma, ¿cómo afecta eso a la charla trivial? ¿Sucede? *(If you’ve had a doctor that doesn’t share your language, how does that affect small talk? Does it happen?)*

6. Cuénteme si es diferente la relacion entre usted y su doctor de cabecera y el doctor que le atendió en el departamento de emergencia. *(Tell me if there was a difference between the relationship between you and your PCP and the doctor who saw you in the emergency department.)*

Probe: ¿Cómo fueron diferentes las conversaciones? *(How were the conversations different?)*

Probe: ¿Le preguntaron diferentes preguntas? *(Did they ask you different questions?)*

Probe: En el departamento de emergencias, ¿le atendió un doctor hispanohablante? *(In the emergency department, were you seen by a Spanish-speaking doctor?)*

Probe: Cuándo usted fue al departamento de emergencia, ¿le hicieron preguntas sobre partes “no medicales” de su vida? ¿Había charla trivial? *(When you went to the emergency department, did they ask you questions about “non-medical” parts of your life? Was there small talk?)*

7. Queremos aprender mas sobre los tipos de preguntas “no medicales” que le hace su doctor, que usualmente se llama la “historia social.” *(We’d like to learn more about the “non-medical” questions that your doctor asks you, sometimes called the “social history.”)*

¿Le preguntó su doctor de cabecera sobre su [responde si/no] *(Did your PCP ask you about [respond yes/no])*

- Ocupación/trabajo *(Work/occupation)*
- Actividades de diversión/pasatiempos/recreación *(Hobbies, pastimes, activities for fun)*
- Dieta *(Diet)*
- Ejercicio *(Exercise)*
- Apoyo social (familia, amigos) *(Social support [friends, family])*
- Seguridad en el hogar *(Safety at home)*
- Vivienda *(Housing)*
- Transporte *(Transportation)*
- Salud mental *(Mental health)*
- Trauma o abuso *(Trauma or abuse)*
- Capacidad para realizar tareas cotidianas *(Activities of daily living)*

Probe: Estabamos hablando del doctor, pero, ¿hay otro personal que le preguntan estas preguntas, como un asistente, una enfermera, una trabajador social, etc? *(We were talking about the doctor, but are there other personnel that ask you these questions, like a medical assistant, nurse, social worker, etc.?)*

Probe: ¿Con que frequencia le hacen estos tipos de preguntas? *(How often do they ask these types of questions?)*

Probe: Cuando usted fue al departamento de emergencia, ¿le hicieron preguntas sobre cualquiera de las preguntas que acabamos de mencionar? *(When you went to the emergency department, did they ask you any of the type of questions we mentioned?*

Probe: ¿Cómo le gustaría que su doctor le preguntara acerca de esta cosas? *(How would you like your doctor to ask you about these things?)*

Probe: ¿Prefiere llenar encuestas o hablar sobre estas temas en persona? *(Do you prefer to fill out questionnaires or talk about these topics in person?)*

Probe: ¿Cuándo cree que no es apropiado o necesario que le hagan estas preguntas? *(When do you think it’s not appropriate or necessary that they ask you these questions?)*

Probe: Aparte de las preguntas de que hemos hablado, ¿usted quisiera que ellos le hicieran otras preguntas “no medicales?” *(Apart from the ones we talked about, would you like for them to ask you other “non-medical” questions?)*

8. [Si no fuera contestado antes] Cuénteme por favor si hubo una diferencia entre como su doctor/medico de cabecera y el doctor de emergencia le pregunto sobre “la historia social.” *([If not answered] Please tell me if there was a difference in how your PCP and the doctor in the emergency department asked you about the “social history.”)*

9. Cuénteme por favor si hubo una diferencia entre como un doctor hispanohablante y un doctor no hispanohablante cuanto le preguntaron sobre “la historia social.” *(Tell me if there has been a difference in how a Spanish-speaking doctor and a non-Spanish-speaking doctor asked you about the “social history.”)*

10. ¿Cambia la relación entre usted y su doctor una vez que le hacen estas preguntas? En caso de si, ¿cómo cambia? *(Does the relationship between you and your doctor change once they ask you*

Probe: ¿Usted opina que es importante que los doctores hagan preguntas sobre “la historia social?” [Podemos repetir la lista si es necesario]. *(Do you think that it’s important for doctors to ask “social history” questions?)*

Probe: ¿Opina que este tipo de comprension fuera de temas “medicales” es importante para el cuidado de salud? *(Do you think that this type of understanding outside of “medical” topics is important to take care of your health?)*

*Appendix A2: English-speaking patient interview guide*

A) Introduction of interviewer and participant

B) Brief overview of the study and the central aims

Summary of study (very simple language): We are doing this study to learn more about how patients develop a good relationship with their doctors. We are interested in how that might be different for patients depending on if they speak the same language as their doctors. We’ll first start by asking questions about how you develop a good or bad relationship with your healthcare team. Then we’ll ask you about how your healthcare team gets to know you as a whole person, beyond your medical issues.

1. Think about the relationship you have with your doctor. Would you say it is a good or bad relationship? Why?

Probe: What does a bad relationship with a doctor look like?

2. Think about all the doctors you’ve had. What could your doctors have done differently to improve their relationship with you?

3. Do you feel like any doctor you’ve worked with knows you well? Why do you feel that way?

Probe: If a doctor wanted to get to know, how could they do that? Are there specific questions a doctor could ask you to get to know you as a person?

Probe: Informal chats (if not discussed earlier): Do you ever discuss non-medical topics with your doctor? What kind of topics do you talk about? When does that happen? How do you think that affects your relationship with your doctor?

4. (Setting: ED/Urgent Care vs. PCP):I want you to think about your primary care doctor versus a doctor you saw in the emergency department. (Provide specific example and date with chief complaint in case patient needs prompting.) How is the relationship different between your primary care doctor and the doctor/(clinician) who saw you in the Emergency Department? How was the conversation different?

5. We’re interested in learning about if and how your doctor gets to know you as a person. One way some doctors do this is ask patients about non-medical parts of their life. Does your regular doctor ask you about non-medical parts of your life? (Example: your hobbies, your values, your home life). (If no): Have other doctors you’ve had asked those types of questions?

6. When you went to the Emergency Department/Urgent Care, did the doctor/clinician there ask you about those non-medical parts of your life? What did they ask you about?

7. Tell me about how you feel about being asked non-medical questions by doctors. Do you think it’s important for them to ask those questions? Do you think this kind of understanding is important for your health care? Would you want them to ask other non-medical things?

8. (Optional if answered in number 3) How would you want your doctor to ask you about these things? When do you think it’s not appropriate or necessary for your doctor to ask you about these things?

9. You mentioned above that your doctor does/doesn’t ask you about non-medical topics. There are different types of non-medical topics. We’d like to know a bit more about the types of non-medical topics patients are asked about. Does your doctor ever ask you about: (List all of them with yes / no)

- Occupation/work
- Activities for fun/hobbies/recreation
- Diet
- Exercise
- Social support (family, friends)
- Safety at home
- Living situation
- Transportation
- Mental health
- Trauma or abuse
- Ability to do daily tasks related to hygiene

Probe: When and how do your doctors ask you about these topics? they usually use a survey, or ask you verbally, or both?

10. Any differences in which questions were asked or how they were asked between your primary care doctor and ones you don’t know as well? Probe: did you get asked any of these questions in the Emergency Department/Urgent Care? Which ones?

Probe: With your regular doctor, when they asked the social history questions, did you feel these questions were necessary? Did you like the way they were asked?

Probe: How, if at all, do you think these questions and the answers to them affect your relationship with your doctor and the care they provide you?

*Appendix A3: Medical interpreter interview guide*

A) Introduction of interviewer and participant

B) Brief overview of the study and its central aims:

C) Our central aims are: to see if and how language barriers and the care setting impact the clinician-patient relationship; if and how language barriers impact the clinicians’ ability to learn more background knowledge about their patient that is not “explicitly medical” in nature; and how both these impacts are reflected in the clinical record.

1. When you imagine a good clinician-patient relationship, what does that look like?

Alternate question: Think about a time when you interpreted and saw some signs of a really good clinician-patient relationship. What were those signs? What about a bad clinician-patient relationship?

2.Informal chatting – when you interpret, what kind of informal chatting have you seen occur between the clinician and the patient?

3. In your experience, what do clinicians ask their patients about that is not “medically related?”

4. As a medical interpreter, tell me your thoughts about how you’ve seen clinical providers and patients build relationships despite not sharing a language.

5. We’ve been discussing good and bad patient-clinician relationships. What about a good, or bad, interpreter-patient relationship? When/how does that happen? How does that impact patient care?

6. In terms of interpretation modality (phone, video, in-person), what modalities of interpretation have you worked in? What modalities, if any, do you prefer? Which modalities, if any, are best for rapport/relationship building during appointments?

7.Urgent care/emergency care vs. Routine appointments w/PCP: What differences do you see in the questions that are being asked to the patient? What about questions that are not necessarily “explicitly medical” in nature?

8. Explain how we are defining contextual understanding: understanding the environment in which someone grows up, plays, works, raises their family, and thrives. This might also include hobbies and interests, as well as someone’s perspective and values about health, and what motivates them to seek different types of care . (Paste this in the chat) Tell me about how you feel about this definition. Would you add anything else or take anything out?

9. Tell me about how you have seen clinicians develop a contextual understanding of their patients.

Alternatively: We’ve been discussing contextual understanding. Based on our definition of contextual understanding, what types of questions have you seen clinicians ask that would add to contextual understanding? Are there things that patients ask/volunteer that would contribute to this? When does this happen during the encounter?

10. This is a list of areas of a patient’s life that might help a clinician get to know more about their patients.

- Occupation/work
- Activities for fun/hobbies/recreation
- Diet
- Exercise
- Social support (family, friends)
- Safety at home
- Living situation
- Transportation
- Mental health
- Trauma or abuse
- Activities of daily living (e.g. ability to do routine daily tasks related to personal care)

Which of the above questions are normally asked the most? The least?

Probe: If asked in a patient intake form, what happens to those answers? Have you ever interpreted an encounter when this type of intake form is not in the patient’s language? Tell me more about that.

Probe: In your experience, who is asking these questions? (E.g. clinician, nurse, MA, social worker, patient health advocate/health navigator?) Does this go into their chart?

11. Are these questions being asked during most patient encounters that you have been a part of? What types of encounters are they asked in (e.g. primary care visit, emergency care, urgent care, inpatient)? Who are the members of the care team who are asking or prioritizing these questions?

12. Do you usually look at patient’s clinical notes or electronic health records? [If yes] do patients usually ask you to help translate their notes?

*Appendix A4: Clinician interview guide*

Interview Guide for Clinicians

A) Introduction of interviewer and participant

B) Overview of study and central aims:

We will first ask you broadly about patient-clinician relationships; then we’ll ask about how knowledge about non-medical information about the patient is collected; and the impact of language discordance, or when you and the patient don’t share a language.

1. I want you to picture a good relationship with a patient. Now a bad one. What distinguishes the two?

Probe: What makes a good vs bad patient-clinician relationship?

Probe: What factors influence if it is a good or bad relationship?

Probe: Do you think you view this differently than a patient? In what way?

2. How do you get to know patients the first time you meet them?

Probe for ED docs: What about a patient with less acuity?

Probe: What impacts whether or not you get to do (______) like you discussed? Do language barriers impact your ability to do (________)? How?

ProbeL What do you like to ask your patients about the first time you meet them in order to get to know them better as people (Non medically related)?

3. Informal chatting – do you like to have more “informal chats or conversations” with patients? (Provide examples) How does that affect your relationship-building/rapport with patients? Do language barriers impact your ability to have those kinds of informal chats with your patients?

4. Types of interpretation: Tell me about your experiences with different modalities of interpretation (phone, video, in-person).

Probe: When are the different modalities used?

Probe: When you compare phone, video, and in-person interpretation, do you think the type of interpretation makes a difference as you’re trying to build a relationship with your patient? If they say yes: how does the type of interpretation affect relationship-building/rapport with your patient?)

Probe: Is there anything you’d like to add about interpretation?

5. Explain how we are defining contextual understanding: understanding the environment in which someone grows up, plays, works, raises their family, and thrives. This might also include hobbies and interests, as well as someone’s perspective and values about health, and what motivates them to seek out different types of care. (copy+paste definition into the chat) Do you think this definition captures non-medical factors that may impact patient care or patient rapport? Would you add anything else or take anything out?

6. Based on how we’ve talked about contextual understanding of a patient, when do you think this kind of information is obtained about a patient?

Probe: Which members of the healthcare team might ask these questions?

Probe: When do you ask these questions?

Probe: Do you ask these questions verbally or using a survey or questionnaire? Which do you prefer?

7. If you document the answers in the EHR, where do you document the answers? (Optional: Some of these topics can be more sensitive than others. How does that influence when you decide to document this information?)

8. How does the clinical setting/appointment type (e.g. Urgent Care, Emergency Department, Outpatient Scheduled Visit) make a difference in taking a social history?

9. What makes it easier or harder to obtain a contextual understanding?

10. This is a list of areas of a patient’s life that might help a clinician get to know more about their patients.

- Occupation/work
- Activities for fun/hobbies/recreation
- Diet
- Exercise
- Social support (family, friends)
- Safety at home
- Living situation/Housing
- Transportation
- Mental health
- Trauma or abuse
- Activities of daily living (e.g. ability to do routine daily tasks related to personal care)

Which of the above are normally asked the most? The least?

11. Do you think language discordance (or when you and the patient speak a different language) affects the ability to get a contextual understanding of the patient? [If yes]: how?

12. Clinical notes piece: Do you notice, as a clinician, differences between the clinical notes of English-speaking patients and non-English-speaking patients? (If yes: Which parts/sections of the notes do you notice a difference?)

Probe: What about when the patient is non-language concordant with their clinician? Have you noticed any differences in those clinical notes? Specific to contextual understanding?

*Appendix Table 1*

| **Patients (n = 13)** |  |
| --- | --- |
| Language Preference |  |
| Spanish | 8 |
| English | 5 |
| Gender |  |
| Woman | 7 |
| Man | 6 |
| Ethnicity |  |
| Hispanic Origin | 9 |
| Non-Hispanic Origin | 4 |
| Race |  |
| Non-Hispanic White | 1 |
| Non-Hispanic Black | 2 |
| Latine | 8 |
| Other | 2 |
|  |  |
| **Clinicians (n = 6)** |  |
| Speak a non-English Language in Clinical Practice |  |
| Yes, Spanish (very good-native speaker) | 4 |
| Yes, Spanish (fair-good) | 2 |
| Gender |  |
| Woman | 4 |
| Man | 2 |
| Ethnicity |  |
| Hispanic Origin | 1 |
| Non-Hispanic Origin | 5 |
| Race |  |
| Non-Hispanic White | 2 |
| Non-Hispanic Black | 0 |
| Latine | 1 |
| Asian | 2 |
| Other | 1 |
| Years of Experience |  |
| 1-10 | 3 |
| 11-20 | 2 |
| 21-30 | 1 |
|  |  |
| **Medical Interpreters (n = 8)** |  |
| Gender |  |
| Woman | 6 |
| Man | 2 |
| Ethnicity |  |
| Hispanic Origin | 2 |
| Non-Hispanic Origin | 6 |
| Race |  |
| Non-Hispanic White | 2 |
| Non-Hispanic Black | 0 |
| Latine | 2 |
| Other | 4 |
| Languages |  |
| Spanish | 3 |
| Chinese Language (Cantonese, Mandarin, Toishanese) | 3 |
| Russian | 2 |
| Years of Experience |  |
| 1-10 | 4 |
| 11-20 | 3 |
| 21-30 | 1 |
